# Online recorded data-based finite-time composite neural trajectory tracking control for underactuated MSVs

**DOI:** 10.3389/fnbot.2022.1029914

**Published:** 2022-10-12

**Authors:** Chunbo Zhao, Huaran Yan, Deyi Gao

**Affiliations:** Merchant Marine College, Shanghai Maritime University, Shanghai, China

**Keywords:** MSVs, trajectory tracking, online recorded data, finite-time control, composite neural networks

## Abstract

This paper presents an online recorded data-based composite neural finite-time control scheme for underactuated marine surface vessels (MSVs) subject to uncertain dynamics and time-varying external disturbances. The underactuation problem of the MSVs was solved by introducing the line-of-sight (LOS) method. The uncertain dynamics of MSVs are approximated by the composite neural networks (NNs). A modified prediction error signal is designed by virtue of online recorded data. The weight updating law of NN is driven by both tracking error and prediction error, introducing additional correction information to the weights of NN, thus improving the learning ability of the NN. Furthermore, disturbance observers can be devised to estimate the compound disturbances consisting of the approximation errors of NNs and external disturbances. Moreover, the smooth function is inserted into the design of the control scheme, and the finite-time composite neural trajectory tracking control of MSVs is achieved. The stability of the MSVs trajectory tracking closed-loop control system is guaranteed rigorously by the Lyapunov approach, and the tracking error will converge to the set of residuals around zero within a finite time. The simulation tests on an MSV verify the effectiveness of the proposed control scheme.

## 1. Introduction

Due to the rapid exploitation of marine resources, marine surface vehicles (MSVs) have been extensively deployed in various fields, such as scientific research applications, commercial cargo transport, missions related to maritime search and maritime emergency rescue (Dai et al., 2015; He and Geng, 2021). The trajectory tracking of MSVs plays a significant and important role in accomplishing different missions at sea (Xiao and Yin, 2018; Zhu et al., 2021b). However, in the complex maritime environment, MSVs will inevitably be affected by unknown external disturbances and uncertain dynamics, which bring great challenges to accurate trajectory tracking control.

In recent years, scholars have proposed fruitful approaches to mitigate the effects of unknown external disturbances and uncertain dynamics, such as neural network (NN) control (Rout et al., 2020; Zhu et al., 2021a), fuzzy control (Wang et al., 2018, 2020), observer-based nonlinear control (Gao and Guo, 2019; Van, 2019; Guo and Zhang, 2020), and the finite-time control (Ning et al., 2020; Wang and Deng, 2020; Zhu et al., 2020). The NNs and fuzzy logic systems are introduced to approximate the uncertain terms of the MSVs, including unmodeled dynamics and unknown dynamics in Wang et al. (2018, 2020), Rout et al. (2020), and Zhu et al. (2021a). In Gao and Guo (2019), Van (2019), and Guo and Zhang (2020), the nonlinear disturbance observers (NDOs) have been devised to estimate the compound uncertainties consisting of model parameter perturbations and unknown disturbances. Combining parameter adaptive technique and backstepping vector design scheme, the unmodeled dynamics of MSVs were addressed in Do (2016) and Ghommam and Saad (2018).

However, most of the literature mentioned above only focuses on the control problem of fully actuated MSVs. In fact, for most MSVs, there are three degrees of freedom, but only two control inputs are available for control, which means that they are underactuated. The methods to solve the problem of underactuation control mainly focus on additional control methods (Do, 2010; Seok Park, 2014; Park and Yoo, 2016), output redefinition control (Shojaei and Arefi, 2015; Zhu et al., 2021c), line-of-sight (LOS) (Shojaei, 2015; Gao et al., 2017; Elhaki and Shojaei, 2021), etc.

The problems of uncertain dynamics and time-varying disturbances deserve further attention, although the above-mentioned literature has yielded fruitful results. The purpose of approximating system uncertainty information with NN has been completed in Xu (2017) and Xu and Sun (2018). To obtain better tracking control performance, uncertain dynamic terms need to be approximated as accurately as possible. By constructing a serial-parallel estimation model, the model prediction error can be obtained in Peng et al. (2016), and it is integrated into the design of the weight update rate of the NN, which effectively improves the transient performance of the system. To reduce the high-frequency oscillations and improve the transient performance of the system, adaptive control modification (Yucelen and Haddad, 2013) and auxiliary filters (Na et al., 2015, 2017; Huang et al., 2018) were introduced to design the adaptive law and NN weight update law, respectively. The error feedback information is embedded into the reference model in Gibson et al. (2013) to reduce the oscillation caused by large gain. The updating law of NNs weights was designed by fusing model prediction errors and the system tracking errors in Xu et al. (2014).

In the above literature, the tracking error of a closed-loop control system can only reach uniformly boundedness. However, tracking errors of MSVs frequently require achieving finite time convergence. Recently, finite-time (FT) control methods have been intensively investigated and adopted for various control systems because of their advantages of fast convergence and strong robustness (Yang et al., 2021, 2022a). For the FT convergence problem, scholars have proposed a large number of advanced FT control techniques, In addition to the sliding-mode-based FT control method, uniformity and the addition of a power integrator (API) are also effective methods to achieve FT stability. Scholars have proposed a large number of effective techniques to achieve FT control, including sliding-mode based FT control methods, homogeneity, and the addition of power integrators (Wang et al., 2017, 2018). The FT trajectory tracking control (Wang et al., 2016) and formation Control (Zhang et al., 2020; Yang et al., 2022b) of the MSVs are well implemented. It can be clearly seen that the control performance of the MSV system is obviously improved under FT control. However, the FT control of MSVs is still a largely open problem suffering from unknown external disturbances and uncertain dynamics.

In this article, we develop an online recorded data-based FT composite neural control scheme for underactuated MSVs suffering from uncertain dynamics and unknown external disturbances. Moreover, the contributions of this article can be listed as follows.

- In this article, an FT composite neural control scheme based on online recorded data is proposed for underactuated MSVs suffering from uncertain dynamics and unknown external disturbances for the first time, and high precision tracking is guaranteed. Compared with the control scheme based on NN, the proposed control scheme can achieve both higher tracking accuracy, faster tracking speed, and a more precise approximation of uncertain dynamics.
- Unlike existing composite learning control schemes, which either converge exponentially (Xu and Shou, 2018; Xu et al., 2019) or in finite time (Pan et al., 2022) but exhibit potential singularity problems, we introduce a smoothing function into the design of the composite learning control scheme such that the tracking errors can achieve FT converge to the neighborhood of zero without singularity.

The remainder of this article is organized as follows. Section 2 introduces the mathematical model of MSV, the problem formulation, some preliminaries, and the principle of NN. Section 3 describes the details of the control scheme design process. The simulation results and comparison are shown in Section 4. Section 5 concludes this paper. Notations: $\tilde{•}$ indicates the error value between • and its estimate $\hat{•}$, which satisfies that $\tilde{•}=•-\hat{•}$.

## 2. Problem formulation

### 2.1. Mathematical model of MSV

The mathematical model of underactuated MSVs moving in the horizontal plane is given by


(1a) $$\begin{array}{l} ẋ=u\cos\varphi -v\sin\varphi \end{array}$$

(1b) $$\begin{array}{l} ẏ=u\sin\varphi +v\cos\varphi \end{array}$$

(1c) $$\begin{array}{l} \dot{\varphi }=r \end{array}$$

(1d) $$\begin{array}{l} \dot{u}=\frac{1}{m_{11}}\left(m_{22}vr-d_{11}u+\tau_{u}+f_{u}+\tau_{eu}\right) \end{array}$$

(1e) $$\begin{array}{l} \dot{v}=\frac{1}{m_{22}}\left(-m_{11}ur-d_{22}v+f_{v}+\tau_{ev}\right) \end{array}$$

(1f) $$\begin{array}{l} ṙ=\frac{1}{m_{33}}\left[\left(m_{11}-m_{22}\right)uv-d_{33}r+\tau_{r}+f_{r}+\tau_{er}\right] \end{array}$$

where (*x, y*) represents the position and φ denotes the orientation of MSVs in the earth-fixed frame. Here, [*u, v, r*]^*T*^ represents surge velocity, sway velocity, and angular rate of MSVs in the body-fixed frame, respectively. The *m*_11_, *m*_22_, and *m*_33_ are nominal values of the inertia mass. The *d*_11_, *d*_22_, and *d*_33_ are hydrodynamic damping parameters. The τ_*eu*_, τ_*ev*_, and τ_*er*_ denote unknown external disturbances. ${\left[f_{u},\;f_{v},\;f_{r}\right]}^{T}$ represents uncertain dynamics including uncertain parts of the model parameters of the MSVs. τ_*u*_ and τ_*r*_ are the control input signal in the surge and yaw directions.

Assumption 1. The unknown external disturbances τ_*ej*_, (*j* = *u, q, r*) satisfies that $|\tau_{ej}|\leq {\bar{l}}_{j}$ and $|{\dot{\tau }}_{j}|\leq {\bar{\tau }}_{ej}$, where ${\bar{l}}_{j}$ and ${\bar{d}}_{j}$ are unknown positive constants.

Assumption 2. The desired trajectory signal *x*_*d*_, *y*_*d*_ and their first two time derivatives are available.

The position error in the body-fixed frame is given


(2a) $$\begin{array}{l} x_{e}=\left(x-x_{d}\right)\cos\varphi +\left(y-y_{d}\right)\sin\varphi \end{array}$$

(2b) $$\begin{array}{l} y_{e}=-\left(x-x_{d}\right)\sin\varphi +\left(y-y_{d}\right)\cos\varphi \end{array}$$

Differentiating (Equations 2a,b) with respect to time yields


(3a) $$\begin{array}{l} {ẋ}_{e}=u+ry_{e}-{ẋ}_{d}\cos\varphi -{ẏ}_{d}\sin\varphi \end{array}$$

(3b) $$\begin{array}{l} {ẏ}_{e}=v-rx_{e}+{ẋ}_{d}\sin\varphi -{ẏ}_{d}\cos\varphi \end{array}$$

Then, we can describe the position error *z*_*e*_ and angle error θ_*e*_ as


(4a) $$\begin{array}{l} z_{e}=z_{s}-z_{0}=\sqrt{x_{e}^{2}+y_{e}^{2}}-z_{0} \end{array}$$

(4b) $$\begin{array}{l} \theta_{e}=\arctan2\left(y_{e},x_{e}\right) \end{array}$$

Together with Equations (2a,b), we can obtain


(5a) $$\begin{array}{l} x_{e}=z_{s}\cos\theta_{e} \end{array}$$

(5b) $$\begin{array}{l} y_{e}=z_{s}\sin\theta_{e} \end{array}$$

A user-design positive constant *z*_0_ is embedded in the design of virtual control law to avoid possible singularity. The main objective of this article is to conceive an online recorded data-based FT composite neural control scheme for underactuated MSVs satisfying Assumptions 1–2 suffering from uncertain dynamics and environmental disturbances tracking the desired trajectory satisfies that tracking errors *z*_*e*_ and θ_*e*_ can converge to a small residual set within a finite time.

### 2.2. Some preliminaries

Lemma 1 (Qi et al., 2020). Consider the nonlinear system $\dot{\varepsilon }=g\left(\varepsilon \right)$, *g*(0) = 0, ε ∈ *R*^*n*^, if Lyapunov function *V*(ε) satisfies that


(6) $$\begin{array}{l} \dot{V}\left(\varepsilon \right)\leq -aV\left(\varepsilon \right)-bV^{J}\left(\varepsilon \right) \end{array}$$

where *a* and *b* are positive constants and 0 < *J* < 1. The system is finite-time stable and there exists a setting time function *T*


(7) $$\begin{array}{l} T\leq \frac{1}{a\left(1-J\right)}\ln\;\frac{aV^{1-J}\left(\varepsilon_{0}\right)+b}{b} \end{array}$$

Lemma 2 (Zhang and Zhang, 2014). For arbitrarily positive constant *f* and 0 < δ < 1, the following inequality always holds.


(8) $$\begin{array}{l} (\overset{n}{\underset{i=1}{\sum }}|f|{)}^{\delta }\leq \overset{n}{\underset{i=1}{\sum }}{\left|f\right|}^{\delta } \end{array}$$

Lemma 3 (Wang and Lin, 2015). For arbitrarily positive constant ϖ and positive constant ϱ, the following inequality always holds.


(9) $$\begin{array}{l} 0<|\varrho |-\frac{\varrho^{2}}{\sqrt{\varrho^{2}+\varpi^{2}}}<\varpi \end{array}$$

### 2.3. Introduction to the principle of radial basis function NN

In general, for any continuous function *L*(*X*) that can be parameterized through the Radial Basis Function NNs with approximation errors ξ, and can be described as


(10a) $$\begin{array}{l} L\left(X\right)=W^{\text{T}}\Phi \left(X\right)+\xi \end{array}$$

(10b) $$\begin{array}{l} \Phi \left(X\right)=exp\left[-{\left(X-c_{h}\right)}^{\text{T}}\left(X-c_{h}\right)/b_{h}^{2}\right],\;h=1,2,\ldots N \end{array}$$

where Φ(*X*) is the basis function vector. *c*_*h*_ and *b*_*h*_ denote the center and the width of the basis function, respectively. *N* represents the number of the node. ξ is the approximation error that satisfies |ξ| ≤ ξ_*m*_ and ξ_*m*_ is an unknown positive constant. Ŵ is the estimate of the *W*. In practice, the uncertain nonlinear function can usually be expressed as $\hat{L}={Ŵ}^{T}\Phi$.

## 3. Details of control law design and stability analysis

Together with Equations (4a) and (5a)-(5b), differentiating *z*_*e*_ with respect to time yields


(11) $$\begin{array}{l} {ż}_{e}=u\cos\theta_{e}+v\sin\theta_{e}+\zeta_{1}\cos\theta_{e}+\zeta_{2}\sin\theta_{e} \end{array}$$

where ζ_1_ and ζ_2_ are expressed as


(12a) $$\begin{array}{l} \zeta_{1}=-{ẋ}_{d}\cos\varphi -{ẏ}_{d}\sin\varphi \end{array}$$

(12b) $$\begin{array}{l} \zeta_{2}={ẋ}_{d}\sin\varphi -{ẏ}_{d}\cos\varphi \end{array}$$

The virtual control law α_*u*_ is designed as


(13) $$\begin{array}{l} \alpha_{u}=\frac{1}{\cos\theta_{e}}(-k_{z1}\frac{z_{e}}{\sqrt{{z_{e}}^{2}+{\iota_{z}}^{2}}}-k_{z2}z_{e}-v\sin\theta_{e}\\ \;-\zeta_{1}\cos\theta_{e}-\zeta_{2}\sin\theta_{e}) \end{array}$$

where *k*_*z*1_, *k*_*z*2_, and ι_*z*_ are positive user-designed constants.

In the surge direction, the velocity error can be expressed as *u*_*e*_ = *u* − α_*u*_. From Equation (13), the Equation (11) can be further rewritten as


(14) $$\begin{array}{l} {ż}_{e}=-k_{z1}\frac{z_{e}}{\sqrt{{z_{e}}^{2}\text{+}{\iota_{z}}^{2}}}-k_{z2}z_{e}+u_{e}\cos\theta_{e} \end{array}$$

In the light of Equations (1d) and (13), the equation for the derivative of *u*_*e*_ is given by


(15) $$\begin{array}{l} m_{11}{\dot{u}}_{e}=m_{22}vr-d_{11}u+\tau_{u}+f_{u}+\tau_{eu}-m_{11}{\dot{\alpha }}_{u} \end{array}$$

The uncertain dynamic of MSVs can be estimated using NN such that $m_{22}vr-d_{11}u+f_{u}=W_{u}^{\text{T}}\Phi_{u}+\xi_{u}$. Here, let *d*_*u*_ = ξ_*u*_ + τ_*eu*_. The ξ_*u*_ is the approximation error of NN and the time derivative of ξ_*u*_ is bound.

In the light of Assumption 1, we have


(16) $$\begin{array}{l} |d_{u}|\leq \Upsilon_{u0},|{\dot{d}}_{u}|\leq \Upsilon_{u} \end{array}$$

where Υ_*u*0_ and Υ_*u*_ are positive constants.

The following error equation is further expressed as


(17) $$\begin{array}{l} m_{11}u_{e}{\dot{u}}_{e}=u_{e}\left(W_{u}^{\text{T}}\Phi_{u}+d_{u}+\tau_{u}-m_{11}{\dot{\alpha }}_{u}\right) \end{array}$$

Then, the following control law is constructed


(18) $$\begin{array}{l} \tau_{u}=-k_{u1}\frac{u_{e}}{\sqrt{{u_{e}}^{2}\text{+}{\iota_{u}}^{2}}}-k_{u2}u_{e}-{Ŵ}_{u}^{\text{T}}\Phi_{u}-{\hat{d}}_{u}+m_{11}{\dot{\alpha }}_{u} \end{array}$$

where *k*_*u*1_, *k*_*u*2_, and ι_*u*_ are positive constants.

Therefore, the Equation (17) can be expressed as


(19) $$\begin{array}{l} m_{11}{\dot{u}}_{e}=-k_{u1}\frac{u_{e}}{\sqrt{{u_{e}}^{2}\text{+}{\iota_{u}}^{2}}}-k_{u2}u_{e}+{\tilde{W}}_{u}^{\text{T}}\Phi_{u}+{\tilde{d}}_{u} \end{array}$$

The prediction error is designed as


(20) $$\begin{array}{l} E_{u}=A_{u}-{Ŵ}_{u}^{\text{T}}p_{u} \end{array}$$

where *p*_*u*_ and *A*_*u*_ are constructed as


(21) $$\begin{array}{l} p_{u}=\int_{t-\tau_{d}}^{t}\Phi_{u}d\tau \end{array}$$

(22) $$\begin{array}{l} A_{u}=\int_{t-\tau_{d}}^{t}(m_{11}{\dot{u}}_{e}+{\hat{W}}_{u}^{\text{T}}\Phi_{u}+{\hat{d}}_{u}+k_{u1}\frac{u_{e}}{\sqrt{{u_{e}}^{2}+{\iota_{u}}^{2}}}\\ \;+k_{u2}u_{e})d\tau \end{array}$$

where τ_*d*_ is an integral interval.

From Equations (21) to (23) can be expressed as


(23) $$\begin{array}{l} E_{u}=\phi_{u}+\int_{t-\tau_{d}}^{t}d_{u}d\tau \end{array}$$

where $\phi_{u}={\tilde{W}}_{u}^{T}p_{u}$.

The composite neural update law can be designed as


(24) $$\begin{array}{l} {\dot{Ŵ}}_{u}=\gamma_{u}\left(u_{e}\Phi_{u}+k_{wu}p_{u}E_{u}-\vartheta_{u}{Ŵ}_{u}\right) \end{array}$$

where γ_*u*_, *k*_*wu*_, ϑ_*u*1_, and ϑ_*u*2_ are positive parameters.

The NDO for the surge direction is designed as


(25a) $$\begin{array}{l} {\hat{d}}_{u}=m_{11}u-\sigma_{u} \end{array}$$

(25b) $$\begin{array}{l} {\dot{\sigma }}_{u}={Ŵ}_{u}^{\text{T}}\Phi_{u}+{\hat{d}}_{u}+\tau_{u}+u_{e} \end{array}$$

where σ_*u*_ is the auxiliary variable.

Using Equations (1d) and (25a)-(25b), taking the time derivative of ${\tilde{d}}_{u}$, we can get


(26) $$\begin{array}{l} {\dot{\tilde{d}}}_{u}={\dot{d}}_{u}-{\tilde{W}}_{u}^{\text{T}}\Phi_{u}-{\tilde{d}}_{u}-u_{e} \end{array}$$

Combining Equation (4b) and (5a)-(5b), taking the time derivative of θ_*e*_


(27) $$\begin{array}{l} {\dot{\theta }}_{e}=-r+\frac{1}{z_{s}}\left(-u\sin\theta_{e}+v\cos\theta_{e}-\zeta_{1}\sin\theta_{e}+\zeta_{2}\cos\theta_{e}\right) \end{array}$$

The virtual control law α_*r*_ can be designed as


(28) $$\begin{array}{l} \alpha_{r}=k_{\theta 1}\frac{\theta_{e}}{\sqrt{{\theta_{e}}^{2}+{\iota_{\theta }}^{2}}}+k_{\theta 2}\theta_{e}+\frac{1}{z_{s}}(-u\sin\theta_{e}+v\cos\theta_{e}\\ \;-\zeta_{1}\sin\theta_{e}+\zeta_{2}\cos\theta_{e}) \end{array}$$

where *k*_θ1_, *k*_θ2_, and ι_θ_ are positive constants.

In the yaw direction, the velocity error can be expressed as *r*_*e*_ = *r*−α_*r*_. From Equation (28), the Equation (27) can be further rewritten as


(29) $$\begin{array}{l} {\dot{\theta }}_{e}=-r_{e}-k_{\theta 1}\frac{\theta_{e}}{\sqrt{{\theta_{e}}^{2}\text{+}{\iota_{\theta }}^{2}}}-k_{\theta 2}\theta_{e} \end{array}$$

In the light of Equations (1f) and (28), the equation for the derivative of *r*_*e*_ is given by


(30) $$\begin{array}{l} m_{33}{ṙ}_{e}=\left(m_{11}-m_{22}\right)uv-d_{33}r+\tau_{r}+f_{r}+\tau_{er}-m_{33}{\dot{\alpha }}_{r} \end{array}$$

The uncertain dynamic of MSVs can be estimated using NN such that $\left(m_{11}-m_{22}\right)uv-d_{33}r+f_{r}=W_{r}^{\text{T}}\Phi_{r}+\xi_{r}$. Here, let *d*_*r*_ = ξ_*r*_ + τ_*er*_. The ξ_*r*_ is the approximation error of NN and the time derivative of ξ_*r*_ is bound.

In the light of Assumption 1, we have


(31) $$\begin{array}{l} |d_{r}|\leq \Upsilon_{r0},|{\dot{d}}_{r}|\leq \Upsilon_{r} \end{array}$$

where Υ_*r*0_ and Υ_*r*_ are positive constants.

The following error equation is further expressed as


(32) $$\begin{array}{l} m_{33}r_{e}{ṙ}_{e}=r_{e}\left(W_{r}^{\text{T}}\Phi_{r}+d_{r}+\tau_{r}-m_{33}{\dot{\alpha }}_{r}\right) \end{array}$$

Then, the following control law is constructed


(33) $$\begin{array}{l} \tau_{r}=-k_{r1}\frac{r_{e}}{\sqrt{{r_{e}}^{2}\text{+}{\iota_{r}}^{2}}}-k_{r2}r_{e}-{Ŵ}_{r}^{\text{T}}\Phi_{r}-{\hat{d}}_{r}+m_{33}{\dot{\alpha }}_{r} \end{array}$$

where *k*_*r*1_ and *k*_*r*2_ are positive constants.

Therefore, the Equation (32) can be expressed as


(34) $$\begin{array}{l} m_{33}{ṙ}_{e}=-k_{r1}\frac{r_{e}}{\sqrt{{r_{e}}^{2}\text{+}{\iota_{r}}^{2}}}-k_{r2}r_{e}+{\tilde{W}}_{r}^{\text{T}}\Phi_{r}+{\tilde{d}}_{r} \end{array}$$

The prediction error is designed as


(35) $$\begin{array}{l} E_{r}=A_{r}-{Ŵ}_{r}^{\text{T}}p_{r} \end{array}$$

where *p*_*r*_ and *A*_*r*_ are constructed as


(36) $$\begin{array}{l} p_{r}=\int_{t-\tau_{d}}^{t}\Phi_{r}d\tau \end{array}$$

(37) $$\begin{array}{l} A_{r}=\int_{t-\tau_{d}}^{t}\left(m_{33}{ṙ}_{e}+{Ŵ}_{r}^{\text{T}}\Phi_{r}+{\hat{d}}_{r}+k_{r1}\frac{r_{e}}{\sqrt{{r_{e}}^{2}\text{+}{\iota_{r}}^{2}}}+k_{r2}r_{e}\right)d\tau \end{array}$$

From Equations (36–38) can be expressed as


(38) $$\begin{array}{l} E_{r}=\phi_{r}+\int_{t-\tau_{d}}^{t}d_{r}d\tau \end{array}$$

where $\phi_{r}={\tilde{W}}_{r}^{T}p_{r}$.

The composite learning update law can be designed as


(39) $$\begin{array}{l} {\dot{Ŵ}}_{r}=\gamma_{r}\left(r_{e}\Phi_{r}+k_{wr}p_{r}E_{r}-\vartheta_{r}{Ŵ}_{r}\right) \end{array}$$

where γ_*r*_, *k*_*wr*_, and ϑ_*r*_ are positive parameters.

The NDO for the yaw direction is designed as


(40a) $$\begin{array}{l} {\hat{d}}_{r}=m_{33}r-\sigma_{r} \end{array}$$

(40b) $$\begin{array}{l} {\dot{\sigma }}_{r}={Ŵ}_{r}^{\text{T}}\Phi_{r}+{\hat{d}}_{r}+\tau_{r}+r_{e} \end{array}$$

where σ_*r*_ is the auxiliary variable.

Using Equations (1f) and (40a)-(40b), taking the time derivative of ${\tilde{d}}_{r}$, we can get


(41) $$\begin{array}{l} {\dot{\tilde{d}}}_{r}={\dot{d}}_{r}-{\tilde{W}}_{r}^{\text{T}}\Phi_{r}-{\tilde{d}}_{r}-r_{e} \end{array}$$

Remark 1. In the view of Equations (24) and (39), the online recorded data-based prediction errors and tracking errors are fused to construct the composite NN weight updating. More information is introduced to construct the weight updating to approximate uncertain dynamics. Hence, the trajectory tracking speed and accuracy of MSVs are improved.

Remark 2. Different from Xu and Shou (2018) and Xu et al. (2019), prediction errors are constructed through online data recording and a smooth function, which realizes the FT converge under the composite neural control scheme based on online recorded data.

Remark 3. *k*_*wu*_ and *k*_*wr*_ in Equations (24) and (39) are designed to enhance the learning competence of the NN. The magnitude of the values of *k*_*wu*_ and *k*_*wr*_ determines whether the values of Ŵ_*u*_ and Ŵ_*r*_ mainly depend on the tracking error or the prediction error.

Remark 4. Combined with the approximation results of uncertain dynamics of MSVs, the NDOs were designed to estimate the lumped disturbances consisting of approximation residuals of NNs and unknown external disturbances. In this article, the developed control scheme guarantees both higher tracking accuracy and a more precise approximation of uncertain dynamics.

The compounded unknown information lumped by the uncertain dynamics of MSVs and unknown external disturbances is represented as *D*_*u*_ and *D*_*r*_.


(42a) $$\begin{array}{l} m_{22}vr-d_{11}u+f_{u}+\tau_{eu}=D_{u} \end{array}$$

(42b) $$\begin{array}{l} \left(m_{11}-m_{22}\right)uv-d_{33}r+f_{r}+\tau_{er}=D_{r} \end{array}$$

Remark 5. It is worth noting that one cannot definitively tell whether ${Ŵ}_{u}^{\text{T}}\Phi_{u}$ and ${Ŵ}_{r}^{\text{T}}\Phi_{r}$ can approximate the *m*_22_*vr* − *d*_11_*u* + *f*_*u*_ and (*m*_11_ − *m*_22_)*uv* − *d*_33_*r* + *f*_*r*_, respectively. Because the NNs and NDOs are sharing each other's information, which means both are sharing “estimation work”. If the estimation ${\hat{D}}_{u}$ and ${\hat{D}}_{r}$ can closely follow the compounded unknown information *D*_*u*_ and *D*_*r*_, respectively, then the purpose of composite neural using NNs and NDOs is realized effectively.

**Theorem 1:** Applying the virtual control laws equation (13), (28), the NN updating laws equation (24), (39), NDOs equation (25a)-(25b), (40a)-(40b) to the MSVs trajectory tracking system (1a)-(1c) and (2a)-(2c) with uncertain dynamics and unknown external disturbances under Assumptions 1–2. Tracking errors can achieve FT converge to the neighborhood of zero. All the signals in MSVs trajectory tracking closed-loop system are uniformly ultimately bounded.

**Proof:** The Lyapunov function can be selected as


(43) $$\begin{array}{l} V=\frac{1}{2}{z_{e}}^{2}+m_{11}u_{e}^{2}+\frac{1}{\gamma_{u}}{\tilde{W}}_{u}^{\text{T}}{\tilde{W}}_{u}+{\tilde{d}}_{u}^{2}+\theta_{e}^{2}+m_{33}r_{e}^{2}\\ \;+\frac{1}{\gamma_{r}}{\tilde{W}}_{r}^{\text{T}}{\tilde{W}}_{r}+{\tilde{d}}_{r}^{2} \end{array}$$

Taking the time derivative of Equation (43), we have


(44) $$\begin{array}{l} \dot{V}=z_{e}{ż}_{e}+m_{11}u_{e}{\dot{u}}_{e}+\frac{1}{\gamma_{u}}{\tilde{W}}_{u}^{\text{T}}\left(-{\dot{Ŵ}}_{u}\right)+{\tilde{d}}_{u}\left(-{\dot{\hat{d}}}_{u}\right)\\ \;+\theta_{e}{\dot{\theta }}_{e}+m_{33}r_{e}{ṙ}_{e}+\frac{1}{\gamma_{r}}{\tilde{W}}_{r}^{\text{T}}\left(-{\dot{Ŵ}}_{r}\right)+{\tilde{d}}_{r}\left(-{\dot{\hat{d}}}_{r}\right) \end{array}$$

Along with Equations (14) and (29), Lemma 3, and Young's inequality, we have


(45) $$\begin{array}{l} z_{e}{ż}_{e}\leq -k_{z1}|z_{e}|-\left(k_{z2}-\frac{1}{2}\right){z_{e}}^{2}+\frac{1}{2}{u_{e}}^{2}+k_{z1}\iota_{z} \end{array}$$

(46) $$\begin{array}{l} \theta_{e}{\dot{\theta }}_{e}\leq -k_{\theta 1}|\theta_{e}|-\left(k_{\theta 2}-\frac{1}{2}\right){\theta_{e}}^{2}+\frac{1}{2}{r_{e}}^{2}+k_{\theta 1}\iota_{\theta } \end{array}$$

In the light of Equations (19) and (34) and Lemma 3, we have


(47) $$\begin{array}{l} m_{11}u_{e}{\dot{u}}_{e}\leq -k_{u1}|u_{e}|+k_{u1}\iota_{u}-k_{u2}{u_{e}}^{2}+u_{e}{\tilde{W}}_{u}^{\text{T}}\Phi_{u}+u_{e}{\tilde{d}}_{u} \end{array}$$

(48) $$\begin{array}{l} m_{33}r_{e}{ṙ}_{e}\leq -k_{r1}|r_{e}|+k_{r1}\iota_{r}-k_{r2}{r_{e}}^{2}+r_{e}{\tilde{W}}_{r}^{\text{T}}\Phi_{r}+r_{e}{\tilde{d}}_{r} \end{array}$$

In view of Equations (24) and (39), we can get


(49) $$\begin{array}{l} -\frac{1}{\gamma_{u}}{\tilde{W}}_{u}^{\text{T}}{\dot{Ŵ}}_{u}=-{\tilde{W}}_{u}^{\text{T}}\left(u_{e}\Phi_{u}+k_{wu}p_{u}E_{u}-\vartheta_{u}{Ŵ}_{u}\right) \end{array}$$

(50) $$\begin{array}{l} -\frac{1}{\gamma_{r}}{\tilde{W}}_{r}^{\text{T}}{\dot{Ŵ}}_{r}=-{\tilde{W}}_{r}^{\text{T}}\left(r_{e}\Phi_{r}+k_{wr}p_{r}E_{r}-\vartheta_{r}{Ŵ}_{r}\right) \end{array}$$

From Equations (26) and (41), we can get


(51) $$\begin{array}{l} {\tilde{d}}_{u}{\dot{\tilde{d}}}_{u}={\tilde{d}}_{u}{\dot{d}}_{u}-{\tilde{d}}_{u}\left({\tilde{W}}_{u}^{\text{T}}\Phi_{u}+{\tilde{d}}_{u}+u_{e}\right) \end{array}$$

(52) $$\begin{array}{l} {\tilde{d}}_{r}{\dot{\tilde{d}}}_{r}={\tilde{d}}_{r}{\dot{d}}_{r}-{\tilde{d}}_{r}\left({\tilde{W}}_{r}^{\text{T}}\Phi_{r}+{\tilde{d}}_{r}+r_{e}\right) \end{array}$$

The Equation (44) can be rearranged as


(53) $$\begin{array}{l} \dot{V}\leq -k_{z1}|z_{e}|-\left(k_{z2}-\frac{1}{2}\right){z_{e}}^{2}-k_{u1}|u_{e}|-\left(k_{u2}-\frac{1}{2}\right){u_{e}}^{2}\\ \;+\vartheta_{u}{\tilde{W}}_{u}^{\text{T}}{Ŵ}_{u}-{\tilde{d}}_{u}^{2}+{\tilde{d}}_{u}{\dot{d}}_{u}-{\tilde{d}}_{u}{\tilde{W}}_{u}^{\text{T}}\Phi_{u}-k_{wu}\phi_{u}\delta_{u}\\ \;-k_{wu}{\phi_{u}}^{2}-k_{\theta 1}|\theta_{e}|-\left(k_{\theta 2}-\frac{1}{2}\right)\theta_{e}^{2}-k_{r1}|r_{e}|\\ \;-\left(k_{r2}-\frac{1}{2}\right){r_{e}}^{2}+\vartheta_{r}{\tilde{W}}_{r}^{\text{T}}{Ŵ}_{r}-{\tilde{d}}_{r}^{2}+{\tilde{d}}_{r}{\dot{d}}_{r}-{\tilde{d}}_{r}{\tilde{W}}_{r}^{\text{T}}\Phi_{r}\\ \;-k_{wr}\phi_{r}\delta_{r}-k_{wr}{\phi_{r}}^{2}+k_{z1}\iota_{z}+k_{u1}\iota_{u}+k_{\theta 1}\iota_{\theta }+k_{r1}\iota_{r} \end{array}$$

where $\delta_{g}=\int_{t-\tau_{d}}^{t}d_{g}d\tau$, *g* = *u, r*. From Equations (16) and (31), ${\delta_{g}}^{2}$ has the supreme expressed as ${\delta_{gm}}^{2}$.

According to Young's inequality, we can get


(54) $$\begin{array}{l} \vartheta_{u}{\tilde{W}}_{u}^{\text{T}}{Ŵ}_{u}\leq \vartheta_{u}\left(-\frac{1}{4}{\tilde{W}}_{u}^{\text{T}}{\tilde{W}}_{u}-\frac{1}{4}{\tilde{W}}_{u}^{\text{T}}{\tilde{W}}_{u}-\frac{1}{4}+\frac{1}{4}+\frac{1}{2}W_{u}^{\text{T}}W_{u}\right)\\ \;\leq -\frac{\vartheta_{u}}{4}{\tilde{W}}_{u}^{T}{\tilde{W}}_{u}-\frac{\vartheta_{u}}{2}{\left({\tilde{W}}_{u}^{\text{T}}{\tilde{W}}_{u}\right)}^{\frac{1}{2}}+\frac{\vartheta_{u}}{2}W_{u}^{\text{T}}W_{u}+\frac{\vartheta_{u}}{4} \end{array}$$

Similarly, we can obtain


(55) $$\begin{array}{l} \vartheta_{r}{\tilde{W}}_{r}^{\text{T}}{Ŵ}_{r}\leq -\frac{\vartheta_{r}}{4}{\tilde{W}}_{r}^{T}{\tilde{W}}_{r}-\frac{\vartheta_{r}}{2}{\left({\tilde{W}}_{r}^{\text{T}}{\tilde{W}}_{r}\right)}^{\frac{1}{2}}+\frac{\vartheta_{r}}{2}W_{r}^{\text{T}}W_{r}+\frac{\vartheta_{r}}{4} \end{array}$$

The Equation (53) can be further rearranged as


(56) $$\begin{array}{l} \dot{V}\leq -k_{z1}\left|z_{e}\right|-(k_{z2}-\frac{1}{2}){z_{e}}^{2}-k_{u1}\left|u_{e}\right|-(k_{u2}-\frac{1}{2}){u_{e}}^{2}\\ \;-\frac{\vartheta_{u}}{2}{\left({{\tilde{W}}_{u}}^{\text{T}}{\tilde{W}}_{u}\right)}^{\frac{1}{2}}-\frac{\vartheta_{u}}{4}{{\tilde{W}}_{u}}^{T}{\tilde{W}}_{u}-{\tilde{d}}_{u}^{2}+{\tilde{d}}_{u}{\dot{d}}_{u}-{\tilde{d}}_{u}{\tilde{W}}_{u}^{\text{T}}\Phi_{u}\\ \;-k_{wu}\phi_{u}\delta_{u}-k_{\theta 1}\left|\theta_{e}\right|-(k_{\theta 2}-\frac{1}{2})\theta_{e}^{2}-k_{r1}\left|r_{e}\right|\\ \;-(k_{r2}-\frac{1}{2}){r_{e}}^{2}-\frac{\vartheta_{u}}{2}{\left({\tilde{W}}_{u}^{\text{T}}{\tilde{W}}_{u}\right)}^{\frac{1}{2}}-\frac{\vartheta_{u}}{4}{\tilde{W}}_{u}^{T}{\tilde{W}}_{u}-{\tilde{d}}_{r}^{2}\\ \;+{\tilde{d}}_{r}{\dot{d}}_{r}-{\tilde{d}}_{r}{\tilde{W}}_{r}^{\text{T}}\Phi_{r}-k_{wr}\phi_{r}\delta_{r}+k_{z1}\iota_{z}+k_{u1}\iota_{u}+k_{\theta 1}\iota_{\theta }\\ \;+k_{r1}\iota_{r}+\frac{\vartheta_{u}}{2}W_{u}^{\text{T}}W_{u}+\frac{\vartheta_{u}}{4}+\frac{\vartheta_{r}}{2}W_{r}^{\text{T}}W_{r}+\frac{\vartheta_{r}}{4} \end{array}$$

According to Young's inequality, Equations (16) and (31), we can get


(57) $$\begin{array}{l} {\tilde{d}}_{u}{\dot{d}}_{u}\leq \frac{1}{2}{\tilde{d}}_{u}^{2}+\frac{1}{2}\Upsilon_{u}^{2} \end{array}$$

(58) $$\begin{array}{l} {\tilde{d}}_{r}{\dot{d}}_{r}\leq \frac{1}{2}{\tilde{d}}_{r}^{2}+\frac{1}{2}\Upsilon_{r}^{2} \end{array}$$

(59) $$\begin{array}{l} -\frac{1}{4}{\tilde{d}}_{u}^{2}\leq -\frac{1}{2}|d_{u}|+\frac{1}{4} \end{array}$$

(60) $$\begin{array}{l} -\frac{1}{4}{\tilde{d}}_{r}^{2}\leq -\frac{1}{2}|d_{r}|+\frac{1}{4} \end{array}$$

Then, the Equation (56) can be further rearranged as


(61) $$\begin{array}{l} \dot{V}\leq -k_{z1}|z_{e}|-\left(k_{z2}-\frac{1}{2}\right){z_{e}}^{2}-k_{u1}|u_{e}|-\left(k_{u2}-\frac{1}{2}\right){u_{e}}^{2}\\ \;-\frac{\vartheta_{u}}{2}{\left({\tilde{W}}_{u}^{\text{T}}{\tilde{W}}_{u}\right)}^{\frac{1}{2}}-\frac{\vartheta_{u}}{4}{\tilde{W}}_{u}^{T}{\tilde{W}}_{u}-\frac{1}{2}|d_{u}|-\frac{1}{4}{\tilde{d}}_{u}^{2}-{\tilde{d}}_{u}{\tilde{W}}_{u}^{\text{T}}\Phi_{u}\\ \;-k_{wu}\phi_{u}\delta_{u}-k_{\theta 1}|\theta_{e}|-\left(k_{\theta 2}-\frac{1}{2}\right)\theta_{e}^{2}-k_{r1}|r_{e}|\\ \;-\left(k_{r2}-\frac{1}{2}\right){r_{e}}^{2}-\frac{\vartheta_{r}}{2}{\left({\tilde{W}}_{r}^{\text{T}}{\tilde{W}}_{r}\right)}^{\frac{1}{2}}-\frac{\vartheta_{r}}{4}{\tilde{W}}_{r}^{T}{\tilde{W}}_{r}-\frac{1}{2}|d_{r}|\\ \;-\frac{1}{4}{\tilde{d}}_{r}^{2}-{\tilde{d}}_{r}{\tilde{W}}_{r}^{\text{T}}\Phi_{r}-k_{wr}\phi_{r}\delta_{r}+k_{z1}\iota_{z}+k_{u1}\iota_{u}+k_{\theta 1}\iota_{\theta }\\ \;+k_{r1}\iota_{r}+\frac{\vartheta_{u}}{2}W_{u}^{\text{T}}W_{u}^{}+\frac{\vartheta_{u}}{4}+\frac{\vartheta_{r}}{2}W_{r}^{\text{T}}W_{r}+\frac{\vartheta_{r}}{4}+\frac{1}{2}\Upsilon_{u}^{2}\\ \;+\frac{1}{2}\Upsilon_{r}^{2}+\frac{1}{2} \end{array}$$

According to Young's inequality and the Lemma 3, we have the following fact


(62) $$\begin{array}{l} -{\tilde{d}}_{u}{\tilde{W}}_{u}^{\text{T}}\Phi_{u}\leq \frac{1}{2}\mu_{u}{\tilde{d}}_{u}^{2}\varpi_{u}^{2}+\frac{1}{2\mu_{u}}{\tilde{W}}_{u}^{\text{T}}{\tilde{W}}_{u} \end{array}$$

(63) $$\begin{array}{l} -\phi_{u}\delta_{u}\leq \frac{1}{2}{\delta_{um}}^{2}+\frac{1}{2}{\phi_{u}}^{2} \end{array}$$

(64) $$\begin{array}{l} -{\tilde{d}}_{r}{\tilde{W}}_{r}^{\text{T}}\Phi_{r}\leq \frac{1}{2}\mu_{r}{\tilde{d}}_{r}^{2}\varpi_{r}^{2}+\frac{1}{2\mu_{r}}{\tilde{W}}_{r}^{\text{T}}{\tilde{W}}_{r} \end{array}$$

(65) $$\begin{array}{l} -\phi_{r}\delta_{r}\leq \frac{1}{2}{\delta_{rm}}^{2}+\frac{1}{2}{\phi_{r}}^{2} \end{array}$$

where μ_*u*_ and μ_*r*_ are positive constants, ||Φ_*u*_|| ≤ ϖ_*u*_ and ||Φ_*r*_|| ≤ ϖ_*r*_.

Therefore, the Equation (61) is further scaled as


(66) $$\begin{array}{l} \dot{V}\leq -k_{z1}\left|z_{e}\right|-(k_{z2}-\frac{1}{2}){z_{e}}^{2}-k_{u1}\left|u_{e}\right|-(k_{u2}-\frac{1}{2}){u_{e}}^{2}\\ \;-\frac{\vartheta_{u}}{2}{\left({\tilde{W}}_{u}^{\text{T}}{\tilde{W}}_{u}\right)}^{\frac{1}{2}}-(\frac{\vartheta_{u}}{4}-\frac{1}{2\mu_{u}}){\tilde{W}}_{u}^{T}{\tilde{W}}_{u}-\frac{1}{2}\left|d_{u}\right|-(\frac{1}{4}\\ \;-\frac{1}{2}\mu_{u}\varpi_{u}^{2}){\tilde{d}}_{u}^{2}-k_{\theta 1}\left|\theta_{e}\right|-(k_{\theta 2}-\frac{1}{2})\theta_{e}^{2}-k_{r1}\left|r_{e}\right|-(k_{r2}\\ \;-\frac{1}{2}){r_{e}}^{2}-\frac{\vartheta_{r}}{2}{\left({\tilde{W}}_{r}^{T}{\tilde{W}}_{r}\right)}^{\frac{1}{2}}-(\frac{\vartheta_{r}}{4}-\frac{1}{2\mu_{r}}){\tilde{W}}_{r}^{T}{\tilde{W}}_{r}-\frac{1}{2}\left|d_{r}\right|\\ \;-(\frac{1}{4}-\frac{1}{2}\mu_{r}\varpi_{r}^{2}){\tilde{d}}_{r}^{2}+k_{z1}\iota_{z}+k_{u1}\iota_{u}+k_{\theta 1}\iota_{\theta }+k_{r1}\iota_{r}\\ \;+\frac{\vartheta_{u}}{2}W_{u}^{\text{T}}W_{u}+\frac{\vartheta_{u}}{4}+\frac{\vartheta_{r}}{2}W_{r}^{\text{T}}W_{r}+\frac{\vartheta_{r}}{4}+\frac{1}{2}\Upsilon_{u}^{2}+\frac{1}{2}\Upsilon_{r}^{2}+\frac{1}{2}\\ \;+\frac{1}{2}k_{wu}{\delta_{um}}^{2}+\frac{1}{2}k_{wu}{\phi_{u}}^{2}+\frac{1}{2}k_{wr}{\delta_{rm}}^{2}+\frac{1}{2}k_{wr}{\phi_{r}}^{2} \end{array}$$

Therefore, we have


(67) $$\begin{array}{l} \dot{V}\leq -aV-hV^{1/2}+b \end{array}$$

where *a* = min{(2*k*_*z*2_ − 1), (2*k*_θ2_ − 1), (2*k*_*u*2_ − 1), (2*k*_*r*2_ − 1), $\left(\frac{1}{2}\vartheta_{u}-\frac{1}{\mu_{u}}\right),$ $\left(\frac{1}{2}\vartheta_{r}-\frac{1}{\mu_{r}}),(\frac{1}{2}-\mu_{u}\varpi_{u}^{2}),(\frac{1}{2}-\mu_{r}\varpi_{r}^{2})\right\}$, *h* = min{2*k*_*z*1_, 2*k*_θ1_, 2*k*_*u*1_, 2*k*_*r*2_, ϑ_*u*_, ϑ_*r*_, 1} and $b=k_{z1}\iota_{z}+k_{u1}\iota_{u}+k_{\theta 1}\iota_{\theta }+k_{r1}\iota_{r}+\frac{1}{2}+\frac{\vartheta_{u}}{2}W_{u}^{\text{*}}W_{u}^{\text{*}}+\frac{\vartheta_{u}}{4}+\frac{1}{2}\Upsilon_{u}^{2}+\frac{\vartheta_{r}}{2}W_{r}^{\text{*}}W_{r}^{\text{*}}+\frac{\vartheta_{r}}{4}+\frac{1}{2}\Upsilon_{r}^{2}+\frac{1}{2}k_{wu}{\delta_{um}}^{2}+\frac{1}{2}k_{wu}{\phi_{u}}^{2}+\frac{1}{2}k_{wr}{\delta_{rm}}^{2}+\frac{1}{2}k_{wr}{\phi_{r}}^{2}$.

From Equation (67), we can obtain


(68) $$\begin{array}{l} \dot{V}\leq -asV-a\left(1-s\right)V-hV^{1/2}+b \end{array}$$

According to Equation (68), if $V>\frac{b}{as}$ we have


(69) $$\begin{array}{l} \dot{V}\leq -a\left(1-s\right)V-hV^{1/2} \end{array}$$

From Lemma 1, *V* converges around $\frac{b}{as}$ within a setting time


(70) $$\begin{array}{l} T\leq \frac{2}{a\left(1-s\right)}\ln\;\frac{a\left(1-s\right)V^{1/2}\left(0\right)+h}{h} \end{array}$$

The theorem has been proved.

## 4. Simulation results and comparison

To validate the superiority of the proposed control scheme in this article, simulation investigations together with comprehensive comparisons of an MSV are addressed in Do and Pan (2004), whereby the main parameters are as follows: *m*_11_$=120\times 10^{3}kg,\;m_{22}=177.9\times 10^{3}kg,\;m_{33}=636\times 10^{5}kg\cdot m^{2}.$ *d*_11_$=215\times 10^{2}kg/s,\;d_{22}=147\times 10^{3}kg/s,\;d_{33}=802\times 10^{4}kg/m^{2}s.$

Next, the performance advantages of the proposed control scheme (FT-ORDCL) are presented through a comprehensive comparison with other neural network-based finite-time control schemes (FT-NN). The simulations are carried out under the proposed trajectory tracking control scheme in the following two cases.

Case 1: Uncertain dynamics of MSV are assumed as ${\left[f_{u},\;f_{v},\;f_{r}\right]}^{T}=$${\left[\left(0.2d_{11}|u|\right)u,\;\left(0.2d_{22}|v|\right)v,\left(0.2|r|\right)r\right]}^{T}$. The unknown external disturbances are assumed as ${\left[\tau_{eu},\;\tau_{ev},\;\tau_{er}\right]}^{T}=$ [10^4^ sin(0.3*t* − π/4) + 10^4^ cos(0.2*t* + π/4) + 2 × 10^4^*N*, 10^3^ sin(0.2*t* − π/4) + 10^3^ cos(0.3*t* − π/4) + 3 × 10^3^*N* · *m*, 10^5^ sin(0.2*t* + π/6) + 10^5^cos(0.5*t* − π/4) − 3 × 10^5^*N* · *m*]^*T*^.

The desired trajectory signal is given as *x*_*d*_ = 200 sin(0.02*t*),*y*_*d*_ = 200 cos(0.02*t*). User-defined parameters for FT-ORDCL and FT-NN control schemes are as follows: [*x*(0), *y*(0), φ(0), *u*(0), *v*(0), *r*(0)] = [20, 190, −0.02π, 0, 0, 0]. *z*_0_ = 10, *k*_*z*1_ = 0.02, *k*_*z*2_ = 0.55, ι_*z*_ = 0.2, *k*_θ1_ = 0.001, ι_θ_ = 0.3, *k*_θ2_ = 0.8, *k*_*u*1_ = 5, ι_*u*_ = 0.2, $k_{u2}=6.5\times 10^{3}$, *k*_*r*1_ = 3, ι_*r*_ = 0.3, $k_{r2}=3.18\times 10^{6}$, γ_*u*_ = 100, γ_*r*_ = 10, *k*_*wu*_ = *k*_*wr*_ = 50, ϑ_*u*1_ = ϑ_*r*1_ = 0.0001, ϑ_*u*2_ = ϑ_*r*2_ = 0.001, τ_*d*_ = 0.05.

**Figure 1:**
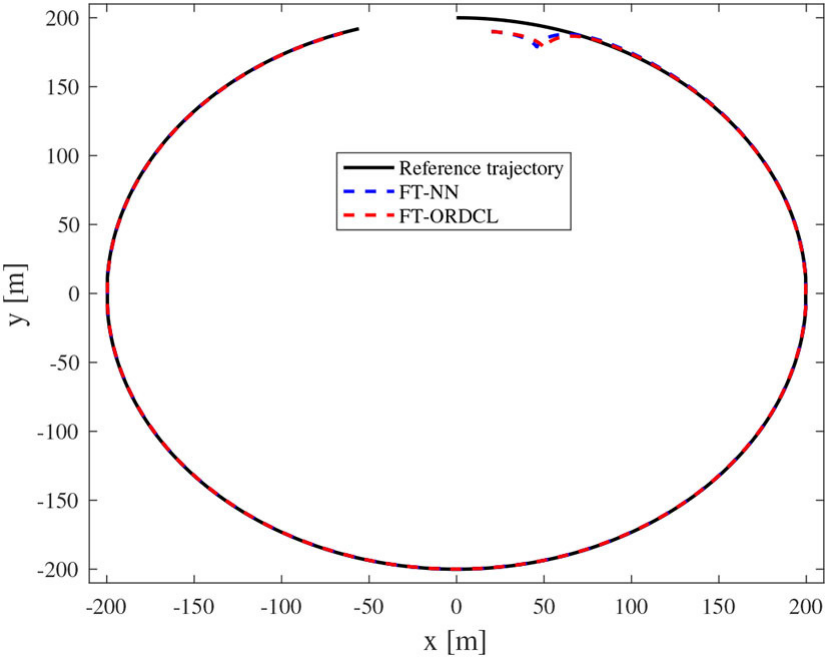
Reference and actual trajectories of the MSV in Case 1.

**Figure 2:**
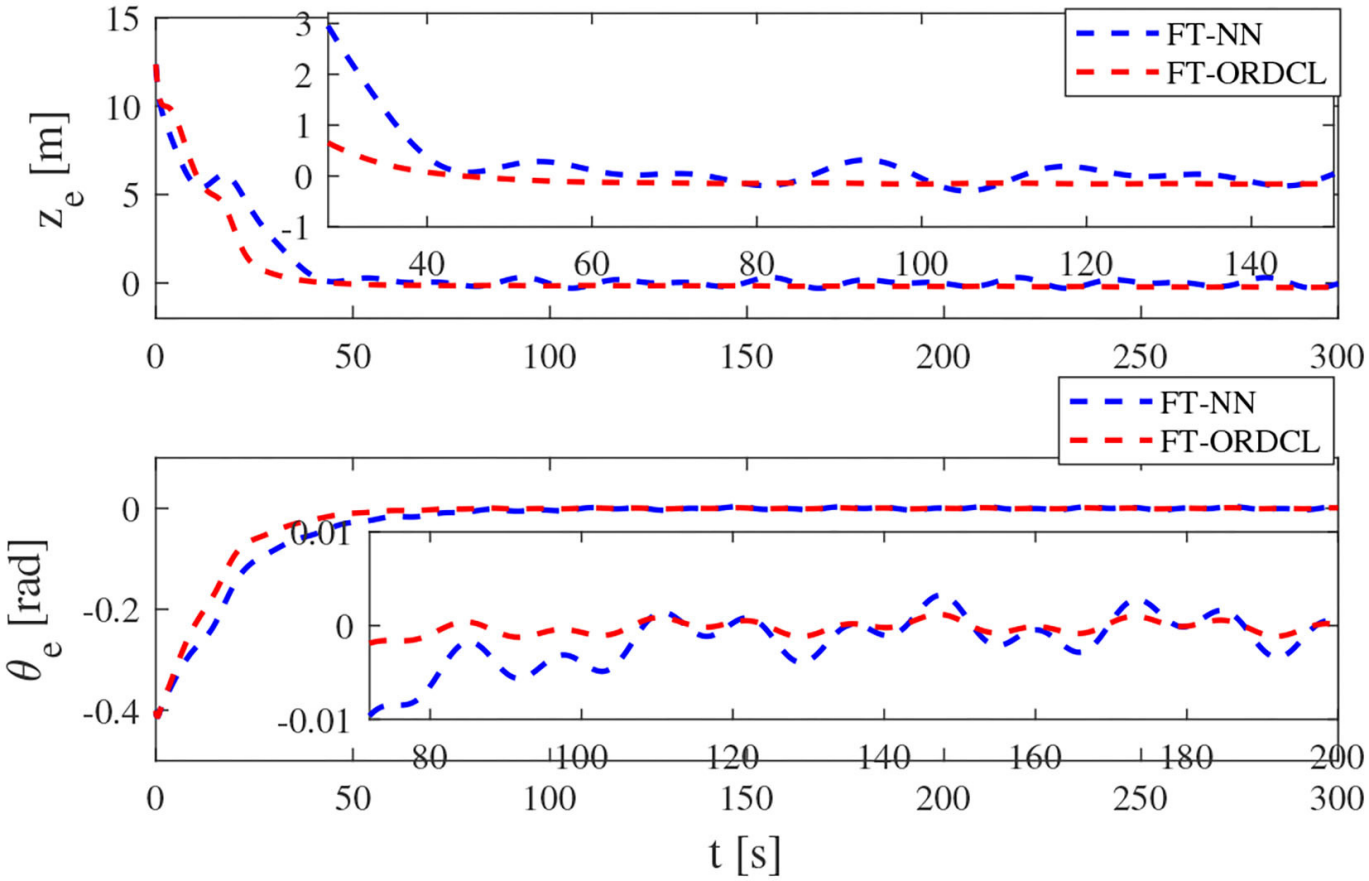
Tracking position error and yaw angle error in Case 1.

**Figure 3:**
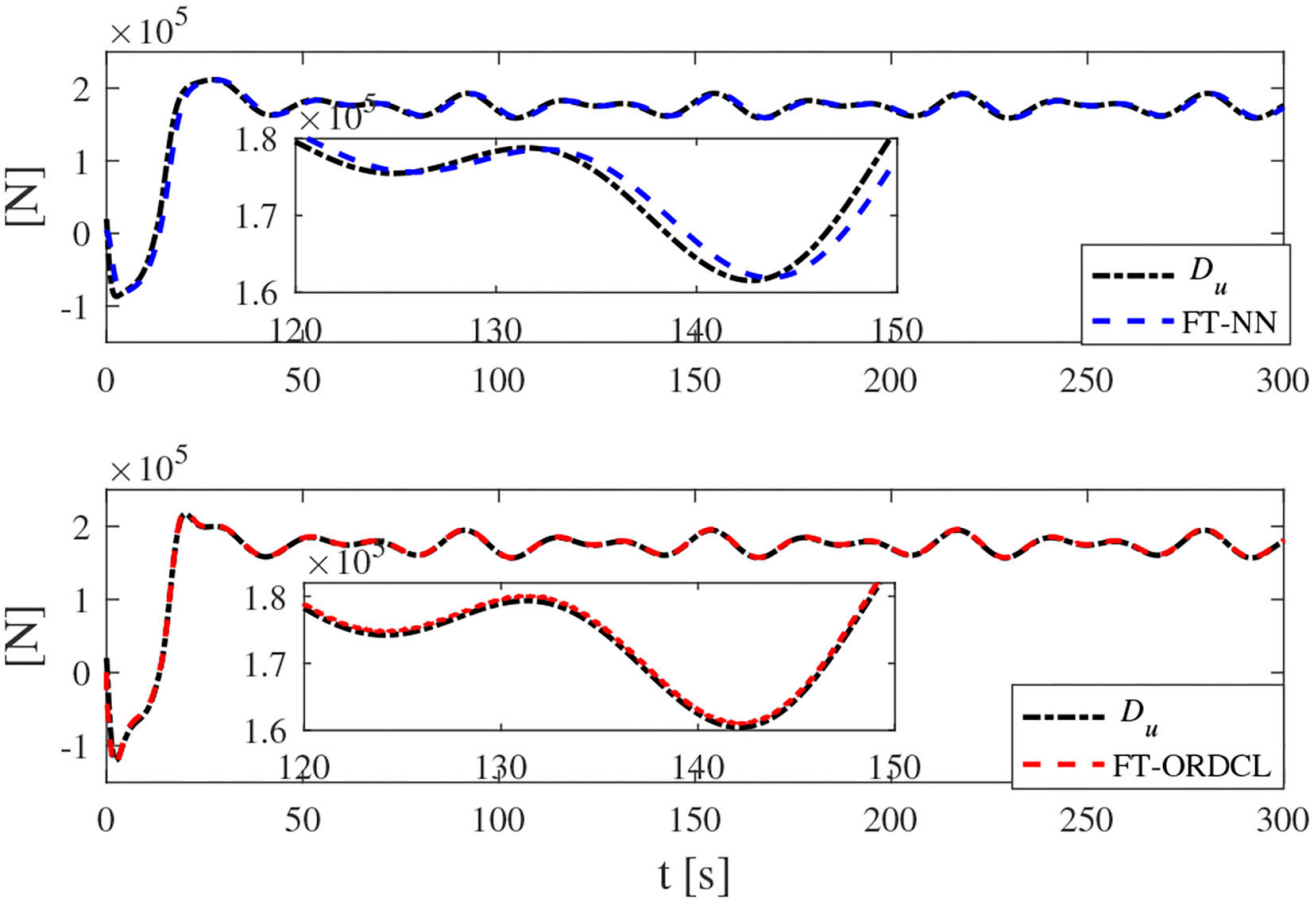
*D*_*u*_ and its estimation in Case 1.

**Figure 4:**
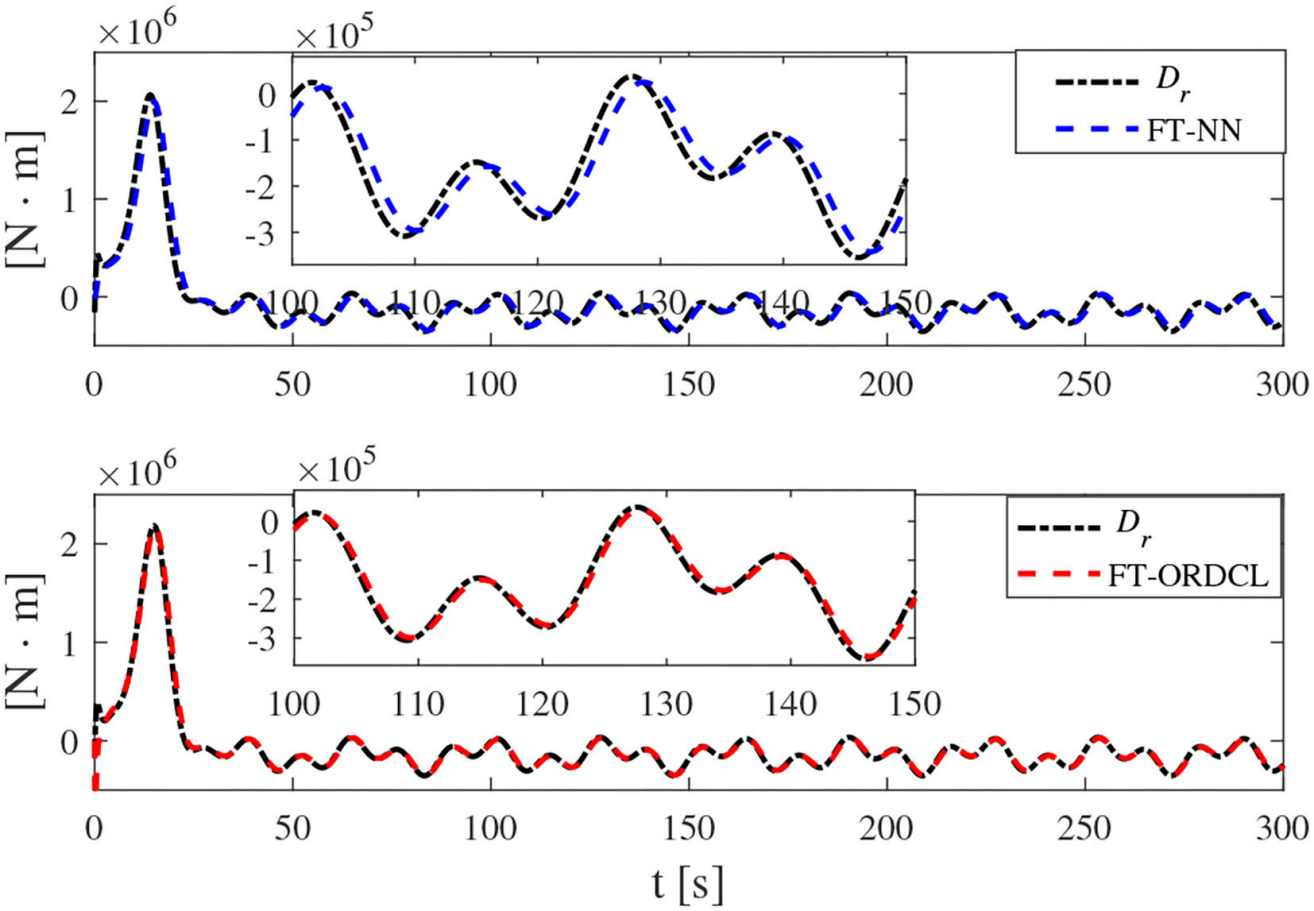
*D*_*r*_ and its estimation in Case 1.

**Figure 5:**
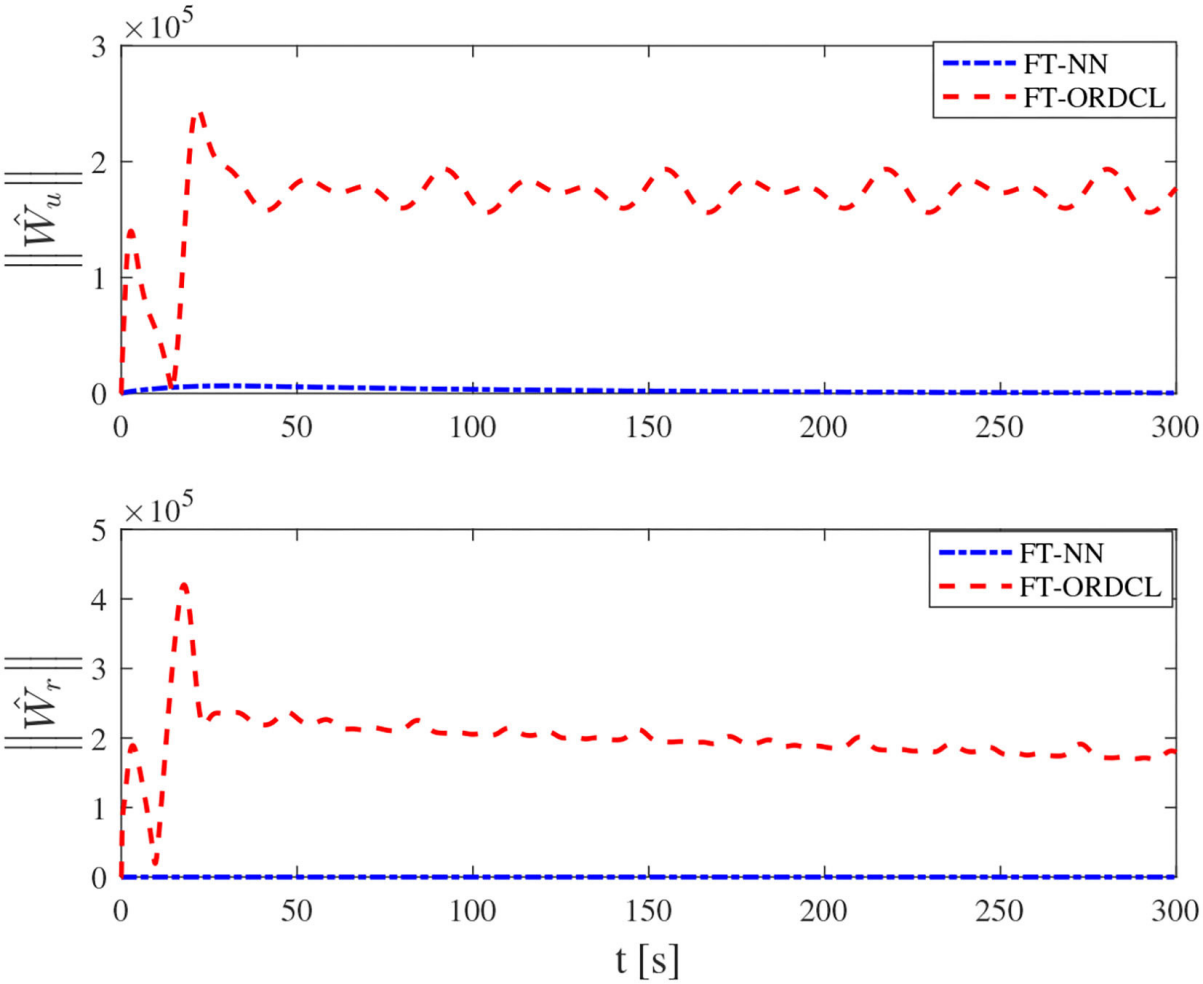
2-norms ||Ŵ_*u*_||, ||Ŵ_*r*_|| of its estimation Ŵ_*u*_ and Ŵ_*r*_ in Case 1.

**Figure 6:**
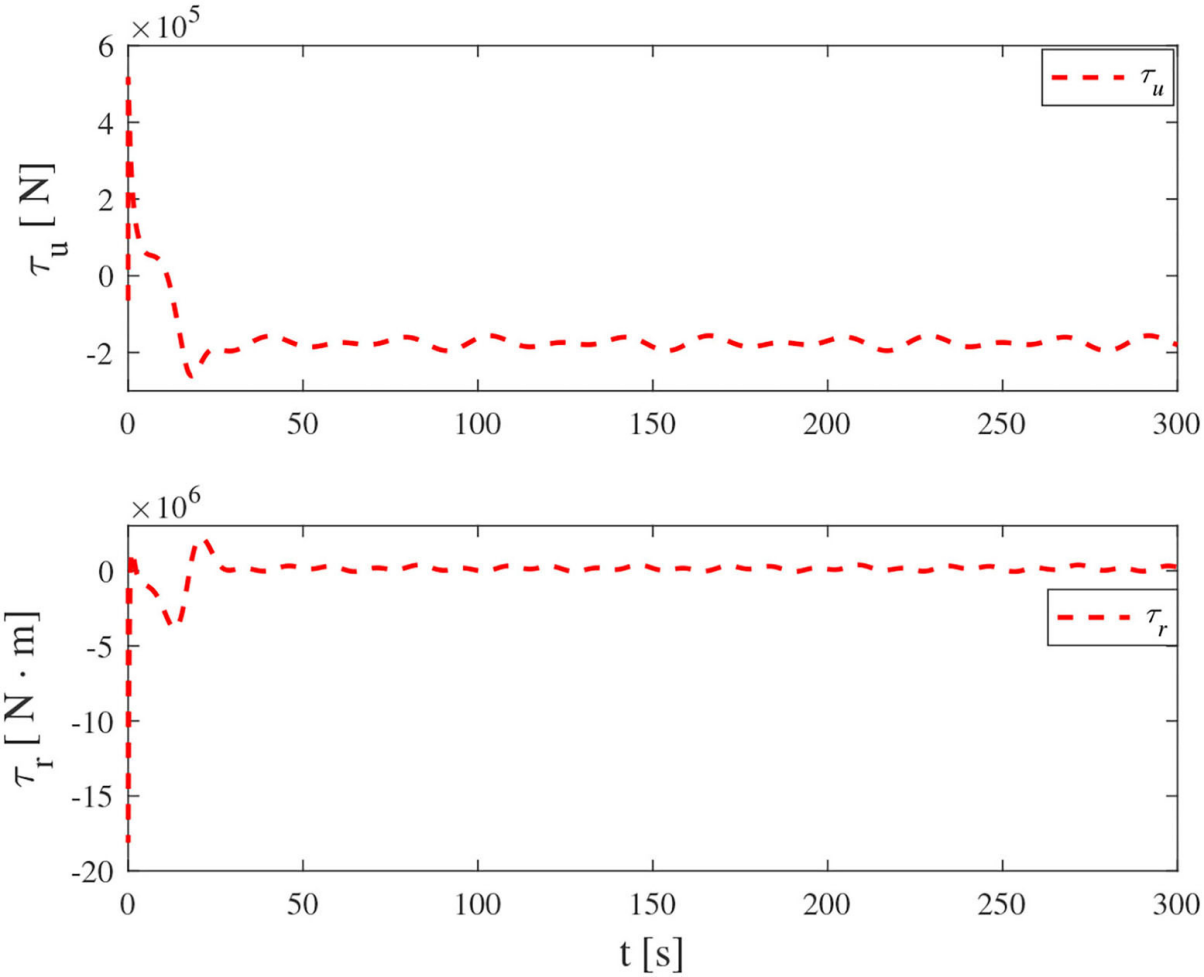
Control input τ_*u*_ and τ_*r*_ in Case 1.

Simulation results under the FT-ORDCL and FT-NN control schemes are illustrated in Figures 1–6. Figure 1 clearly shows that the desired trajectory can be tracked under uncertain dynamics and time-varying disturbances under both control schemes. From Figure 2, the results show that FT-ORDCL can accomplish faster convergence and more accurate tracking of desired trajectories than FT-NN. The approximate results of unknown information are clearly shown in Figures 3, 4, thus further supporting the conclusion in Figure 2. The estimated value of 2-norms of the NN weights are bounded and reasonable as seen in Figure 5. The control force τ_*u*_ and control torque τ_*r*_ are plotted in Figure 6. From a practical point of view, the control force and control torque are bounded and reasonable.

Case 2: The unknown dynamics are increased by +10% and the bigger unknown disturbance is given as ${\left[\tau_{eu},\;\tau_{ev},\;\tau_{er}\right]}^{T}=$ [1.2 × 10^4^ sin(0.3*t* − π/4) + 1.2 × 10^4^ cos(0.2*t* + π/4) + 2.4 × 10^4^*N*, 1.2 × 10^3^ sin(0.2*t* − π/4) + 1.2 × 10^3^ cos(0.3*t* − π/4) + 3.6 × 10^3^*N* · *m*, 1.2 × 10^5^ sin(0.2*t* + π/6) + 1.2 × 10^5^ cos(0.5*t* − π/4) − 3.6 × 10^5^*N* · *m*]^*T*^. The design parameters and the initial conditions are given the same as those in Case 1.

**Figure 7:**
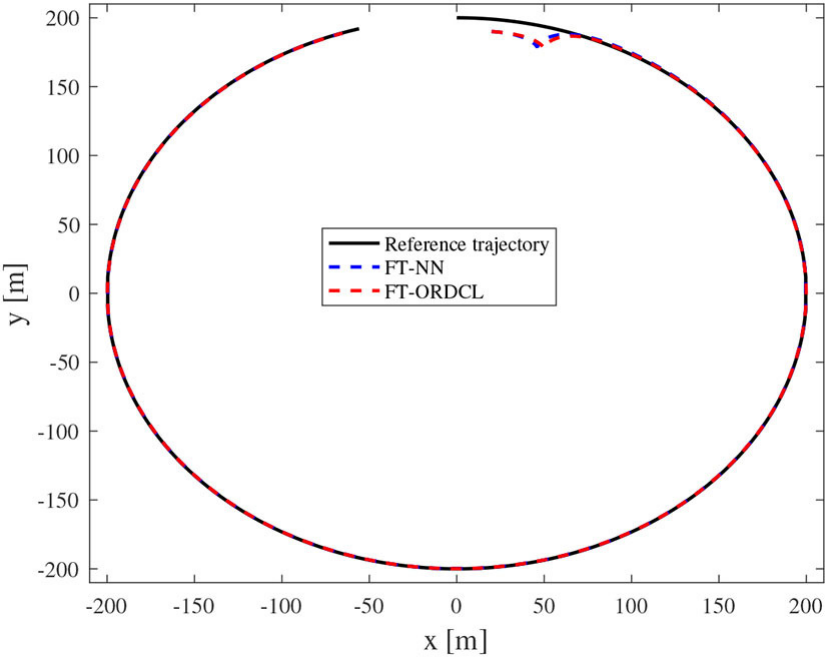
Reference and actual trajectories of the MSV in Case 2.

**Figure 8:**
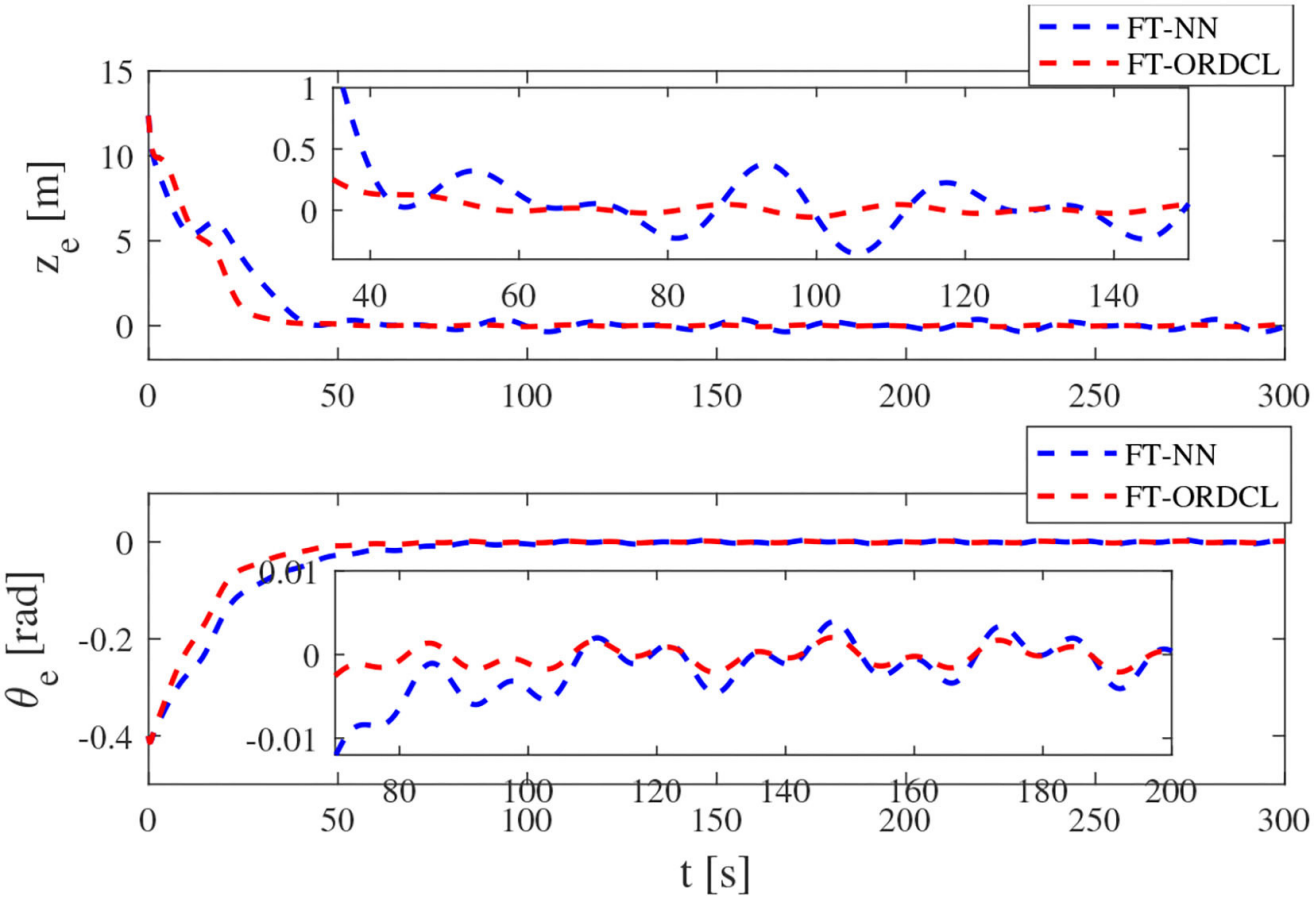
Tracking position error and yaw angle error in Case 2.

**Figure 9:**
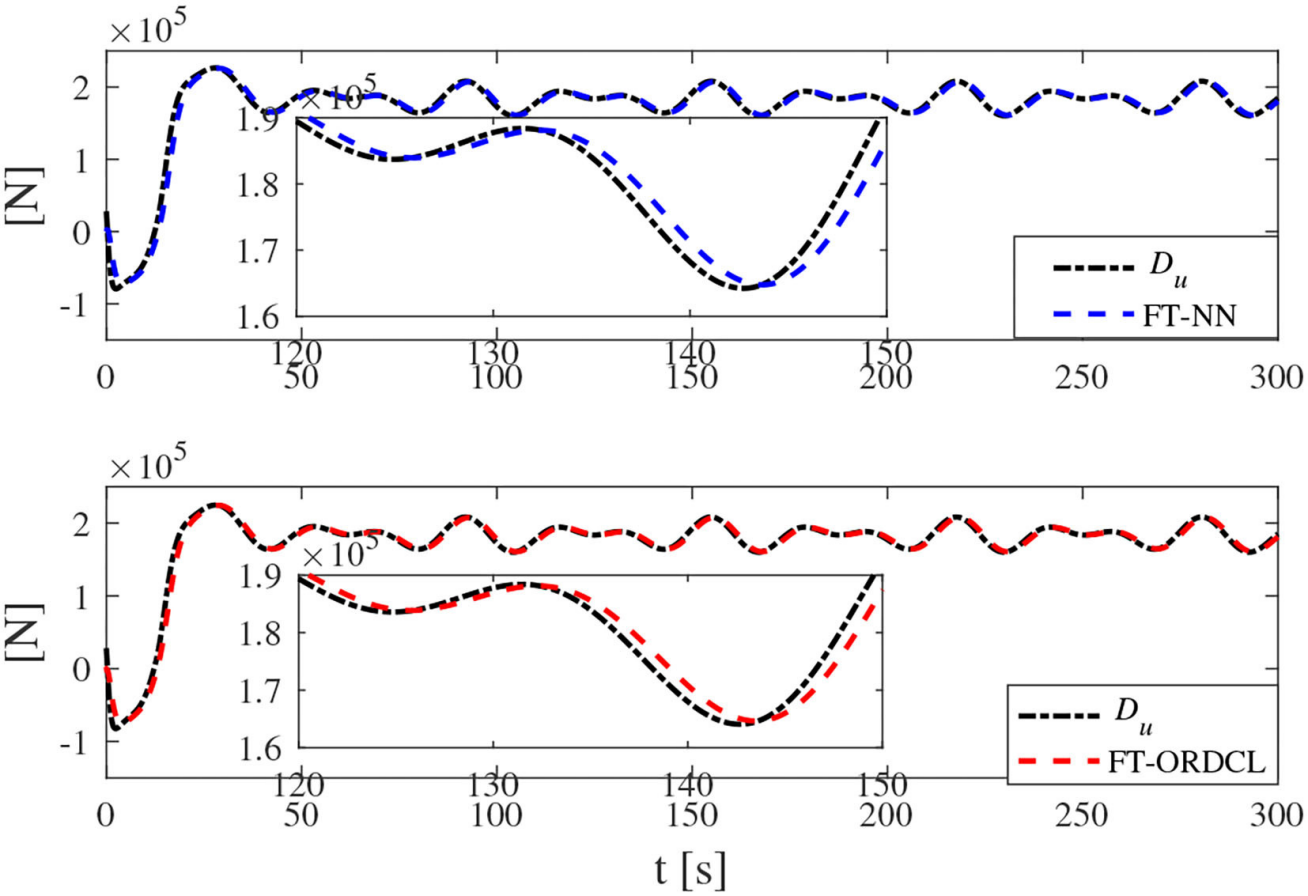
*D*_*u*_ and its estimation in Case 2.

**Figure 10:**
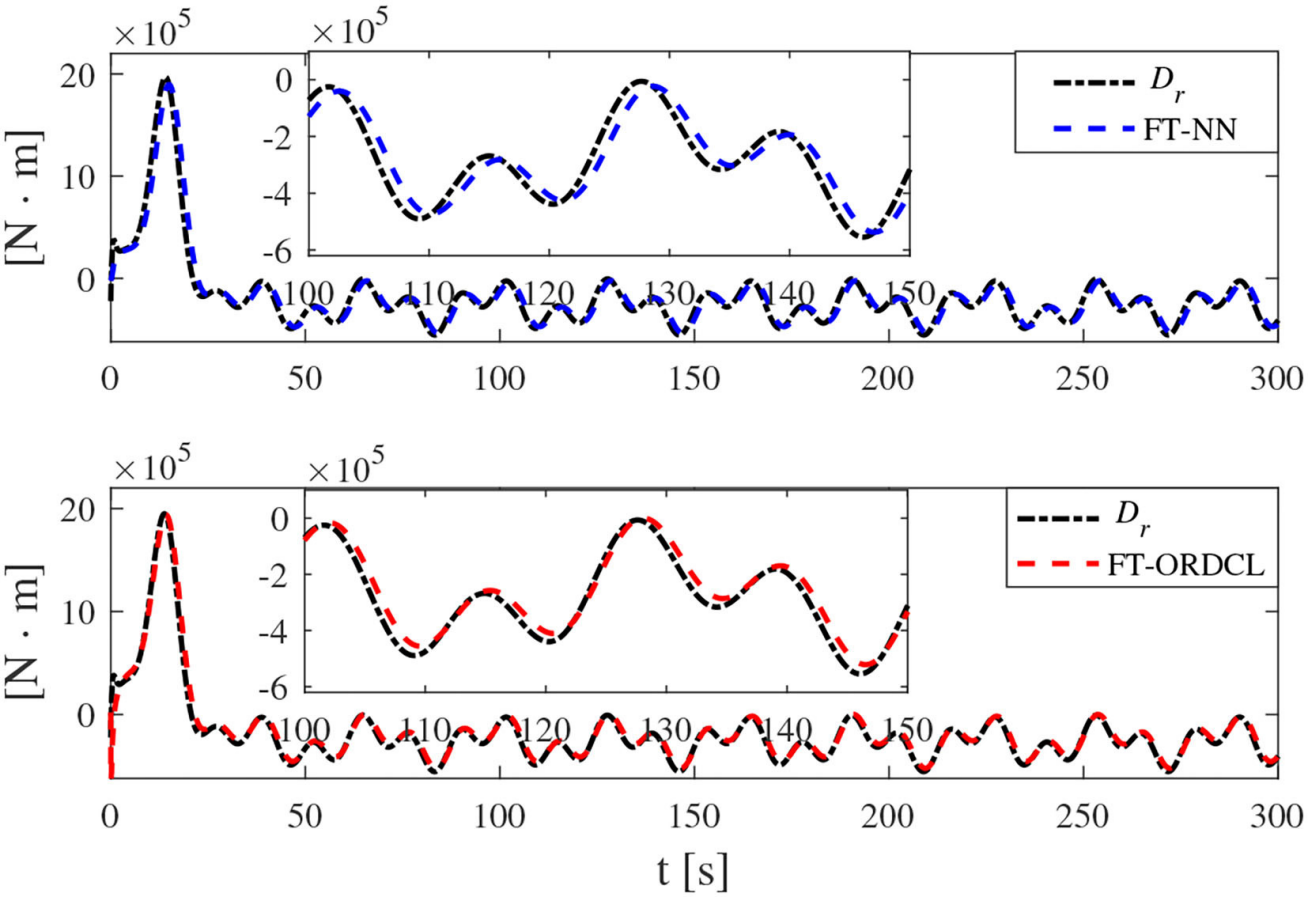
*D*_*r*_ and its estimation in Case 2.

**Figure 11:**
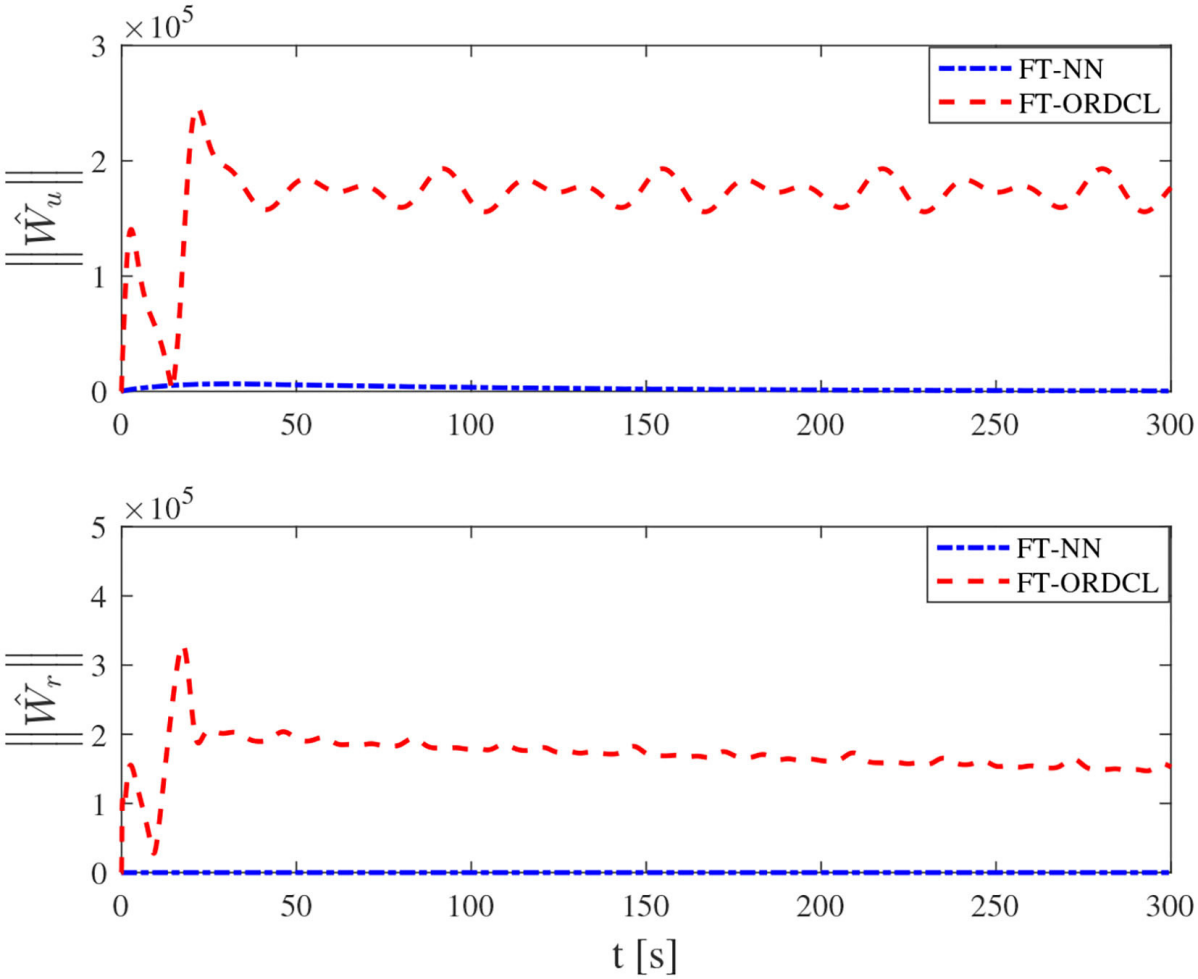
2-norms ||Ŵ_*u*_||, ||Ŵ_*r*_|| of its estimation Ŵ_*u*_ and Ŵ_*r*_ in Case 2.

**Figure 12:**
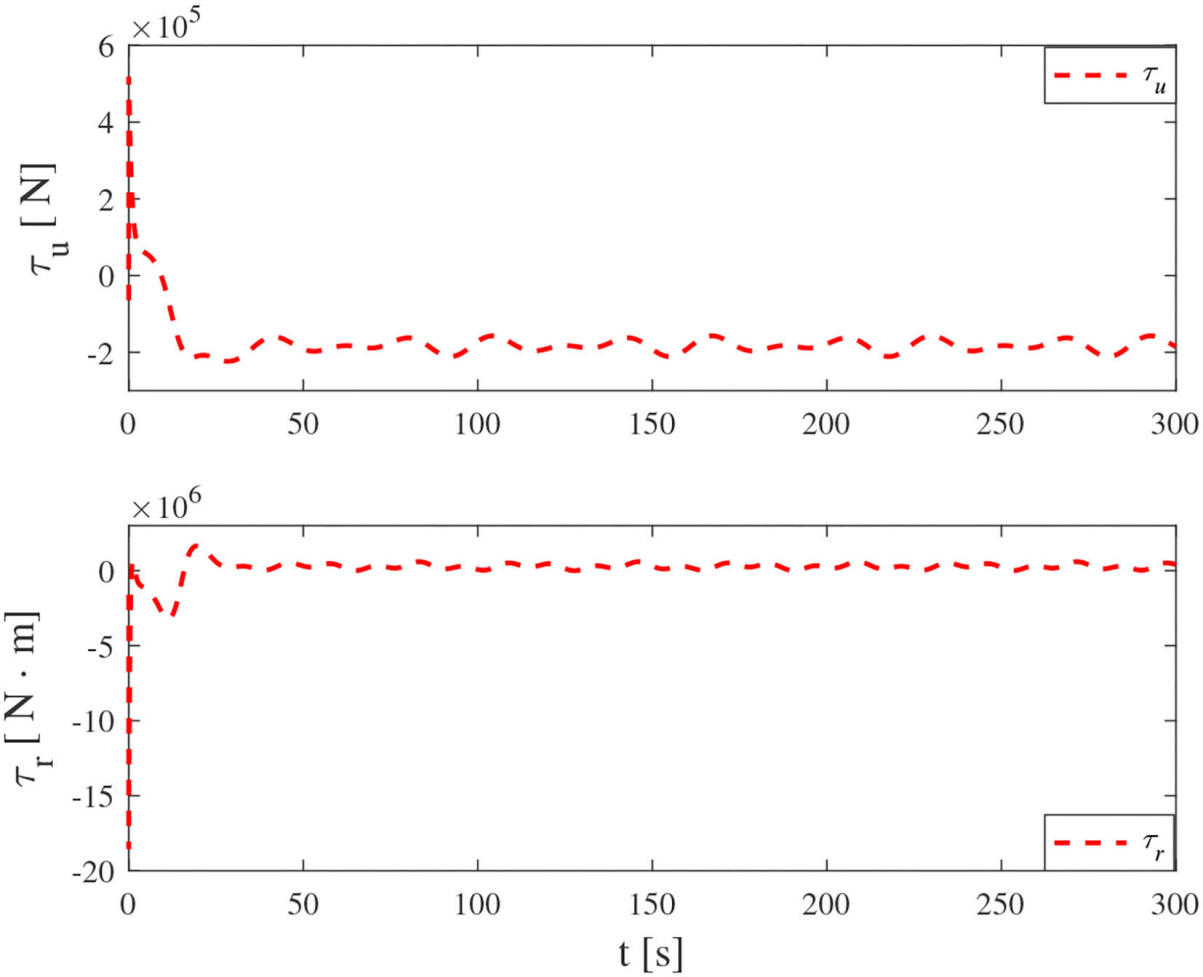
Control input τ_*u*_ and τ_*r*_ in Case 2.

Simulation results under the FT-ORDCL and FT-NN control schemes in Case 2 are provided in Figures 7–12. It is clearly depicted from Figure 7, that MSVs can track the desired trajectory in the presence of uncertain dynamics and time-varying ocean disturbances under both control schemes in Case 2. From Figure 8, the results show that FT-ORDCL can accomplish faster convergence and more accurate tracking of desired trajectories than FT-NN. It can be seen from Figures 9, 10, the same conclusion can be obtained in Case 1. The proposed control scheme has good adaptability and robustness. The estimated value of 2-norms of the NN weights are bounded as seen in Figure 11. The control force τ_*u*_ and control torque τ_*r*_ are plotted in Figure 12. From Figure 12, the corresponding control inputs are bounded and reasonable.

## 5. Conclusion

In this article, the problem of FT trajectory tracking control for underactuated MSVs, which suffer from uncertain dynamics and unknown external disturbances, has been solved by devising a composite neural control scheme based on online recorded data. The uncertain dynamics and unknown external disturbances were compensated exactly by the composite NNs based on online recorded data and the NDOs, respectively. By virtue of the LOS approach, the underactuation problem of the MSV is addressed. A smooth function is inserted into the design of the proposed control scheme artistically, and the FT trajectory tracking control of MSVs is realized based on online data recording composite NNs. The comparison of simulation results and methods shows the effectiveness and superiority of the developed control scheme.

Furthermore, the developed control scheme in this article can be extended to the trajectory tracking control of multiple-input multiple-output or single-input single-output systems with unknown external disturbances and uncertain dynamics. For extensions to the existing study, the proposed control scheme can be combined with fault-tolerant control and event-triggered schemes to achieve more complex control objectives.

## Data availability statement

The datasets presented in this study can be found in online repositories. The names of the repository/repositories and accession number(s) can be found in the article/supplementary material.

## Author contributions

CZ: conceptualization and investigation. HY: writing and conceptualization. DG: methodology and formal analysis. All authors contributed to the article and approved the submitted version.

## Funding

This study was supported by the National Science Foundation of China under Grant No. 52071201.

## Conflict of interest

The authors declare that the research was conducted in the absence of any commercial or financial relationships that could be construed as a potential conflict of interest.

## Publisher's note

All claims expressed in this article are solely those of the authors and do not necessarily represent those of their affiliated organizations, or those of the publisher, the editors and the reviewers. Any product that may be evaluated in this article, or claim that may be made by its manufacturer, is not guaranteed or endorsed by the publisher.
